# Development of a Valid and Reliable Questionnaire to Identify Professional Opinion Regarding Organ Transplantation System

**Published:** 2017-08-01

**Authors:** B. Sah, A. Ayer, B. N. Yadav, S. Jha, S. K. Yadav

**Affiliations:** 1 Department of Forensic Medicine and Toxicology, B. P. Koirala Institute of Health Sciences, Dharan, Nepal; 2 Department of Conservative Dentistry & Endodontics, B. P. Koirala Institute of Health Sciences, Dharan, Nepal; 3 Institute of Liver Transplant and Regenerative Medicine. Medanta, The Medicity, New Delhi (NCR), India

**Keywords:** Tissue and organ procurement, Directed tissue donation, Organ transplantation, Surveys and questionnaires, Reproducibility of results

## Abstract

**Background:**

Currently, the Nepalese law permits organ donation by an individual who falls into the category of a “close relative” of the recipient. There is a need for expansion of the live organ donor pool beside close relatives. Different systems of organ transplantation are followed by several countries and the professional opinions that underpin these systems need to be studied.

**Objective:**

To generate a questionnaire related to different organ transplant systems and validate it so that it can be used to collect mass professional opinions.

**Methods:**

Item generation, item reduction, item scaling, and pretesting were used to develop a questionnaire. The final version of the questionnaire was reviewed by experts for its content validity and then was used twice for participants at a 20-day interval to calculate Cronbach’s alpha for testing its internal consistency and Intra-class correlation for testing its test and retest reliability.

**Results:**

The questionnaire was found to be valid and reliable with an overall Cronbach’s alpha of 0.701. Intra-class correlation scores for each question in both test and retest were correlated.

**Conclusion:**

A valid and reliable questionnaire was developed that can be used to collect mass professional opinions to assist policy makers to establish a better organ transplant system.

## INTRODUCTION

Organ transplantation is the current standard of care for end-stage organ damage. So, the laws regulating organ transplantation must be supportive and encouraging for the organ donors, recipients, and health professionals. In the USA alone, more than 120,000 patients are on the waiting list; on average, 22 people die each day while waiting for a transplant [1]. The median waiting time for deceased donor renal transplant ranges from 0.61 to 4.57 years in the USA and is expected to increase further annually [2, 3]. There is a huge demand for organ transplantation in Asian countries with China having waiting list of around 1.5 million people [4].

Many countries have already amended the human organ transplantation laws while some countries are in the process of amendment of laws to meet their increased demand for organs. Several European countries have presumed consent systems in which the wishes of the deceased’s family is taken into consideration [5]. The Spanish system includes a component called, “active detection”—transplant coordinators in each hospital consult with family members of patients in intensive care, advising them to support donation [5].

Explicit opt-out systems presume consent unless an individual expresses their refusal to become a potential donor [6]. This system is currently being practiced in countries like Austria, Belgium, the Czech Republic, Finland, France, Greece, Hungary, Israel, Italy, Luxembourg, Norway, Poland, Slovenia, Spain, Sweden, and Turkey, which has made their rate of organ donation 25% to 30% higher than those countries needing explicit consent [7]. Some Asian countries have expanded the donor’s eligibility criteria whereas others have even accepted the provision of donor’s compensations (Table 1).

**Table 1:** Living donor transplantation policies in selected Asian countries by donor restriction and compensation. (Original data from Jingwei AH, *et al*. [8])

Countries	Donor Restriction			Donor Compensation	
	Living Related		Living Unrelated	Kidney	Liver
	Close relatives	Emotionally attached			
Nepal	Yes	No	No	—	—
Taiwan	Yes	No	No	—	—
Hong Kong	Yes	No	No	—	—
China	Yes	Yes	No	—	—
India	Yes	Yes	No	—	—
Malaysia	Yes	Yes	No	—	—
Iran	Yes	Yes	Yes	US$ 2,000-4,000	N/A
Japan	Yes	Yes	Yes	—	—
Korea	Yes	Yes	Yes	—	—
Philippines	Yes	Yes	Yes	—	—
Saudi Arabia	Yes	Yes	Yes	US$ 13,300 and other benefits	
Singapore	Yes	Yes	Yes	Still in discussion	

In 1987, Singapore passed the Human Organ Transplant Act, which applies the priority rule with an opt-out system [9]. This means those not registered as non-donors will be entitled to priority in the allocation of organs for transplantation purposes. Under its laws, those between 21 and 60 years old who die in accidents are assumed to be kidney donors unless they opt out. Older people as well as Muslims are in a separate tier, and they must opt in [10]. Chile also started the priority rule with the opt-out system by amending the law in October 2013 [11]. The Ministry of Health (MOH) of Singapore in November 2008 proposed that paired matching for the exchange of organs be allowed in Singapore to increase the chances of improved transplant outcomes and to save more lives [8]. Under this arrangement, patients can exchange donors mutually.

In 2006, Iran became the only country in Asia to legitimize free kidney sale, which leads Iran to third place globally in living kidney donation rates [12]. As per World Health Organization (WHO), the government of Saudi Arabia in October 2007 passed a law to pay a monetary “reward” of US$ 13,300 and other benefits, including lifetime medical care, for unrelated organ donors in a system regulated at the national level. This law has quadrupled the rates of living kidney donation in Saudi Arabia within a short period [12].

The regulatory system in China is relatively lagging behind its medical development, which has allowed actual organ business to exist in China [13]. Nepal too, has liver and heart transplant surgeons but they cannot practice in the absence of specific laws. Organ transplantation in Asia is usually regarded as a policy issue, rather than a clinical issue, but Malaysia is an exception to this consideration. Malaysia has no laws regulating living donation and in the absence of laws, living donation is presumed to be legally permissible under valid donor’s consent [8].

Nepali laws have restricted live organ donation only among close relatives, that is son, daughter, mother, father, brother, sister, uncle, nephew, niece, grandfather, grandmother from the father’s side; grandson, grand-daughter from the son’s side; grandson, grand-daughter from the daughter’s side; and includes husband, wife, adopted son, adopted daughter, stepmother, stepfather, father-in-law, mother-in-law, with whom relationship has constantly existed since two years ago [14]. The Ministry of Health and Population estimates Nepal can currently only meet 1% of the demand for kidney transplantation; there is no facility for liver, pancreas, bone marrow, heart or lung transplantation in the country [15]. This indicates the need to learn from the countries with higher donation rate and modify our existing laws as well as formulate some new laws.

As transplant services depend on public support, not only due to high costs, but also because the essential prerequisite for solid organ transplantation is a sufficient number of organ donors, the public conceptions of what a transplant service should aim for are of substantial interest [16]. The main objective of this research was therefore to generate a questionnaire related to different organ transplant systems and validate it so that it can be used to collect professional and public opinions.

## MATERIALS AND METHODS

To develop a questionnaire, we performed item generation, item reduction, item scaling, and pretesting as outlined below. We then tested its validity and reliability in 153 subjects above 18 years who gave written informed consents. Ethical approval was obtained from the Institutional Review Committee (B.P. Koirala Institute of Health Sciences).

Item Generation

Literature related with laws regulating organ transplant systems of different countries and of Nepal was reviewed. The points lacking in Nepali Laws and the points that are present but needing updates were used as a template from which questions were developed. Colleagues were also consulted to include unpublished important items. The second step involved interviewing colleagues with expertise in the field of health-related laws and organ transplantation. Finally, patients and their relatives were informally interviewed about the difficulties they faced or are facing on organ transplantation.

Item Reduction

A focus group consisting of the principal questionnaire developer, a forensic expert, a liver transplant surgeon, a cardio-thoracic surgeon, a dental surgeon, and a statistician reviewed the items generated. They decided that three domains—increase rate of organ donation (IROD) domain, decrease rate of donation-related crime (DRDC) domain, and ethical and legal issue related with donation (ELID) domain were necessary.

Item Scaling

Each answer was given a score in decreasing order—maximum for a most expected answer (i.e., answer supporting organ donation) and minimum for the least expected answer.

Face/Content Validity

Six forensic experts and two transplant surgeons were given the questionnaire to review its contents and comment on its overall comprehensibility, specifically, on any questions they would add, delete, or modify. One suggestion was to add a question regarding priority criteria for receiving an organ. All the experts validated the questions with minor corrections for some questions.

## RESULTS

Pretesting

Pretesting was done on 4^th^ year Bachelor of Medicine and Bachelor of Surgery, and Bachelor of Dental Surgery students (10% of total sample size), with interaction on each question, showed the questionnaire to be reliable for testing and retesting.

Reliability

Testing was done in all participants, without any interaction, showed the questionnaire was reliable with an overall Cronbach’s α of 0.701; this value did not increase after deleting any items. Domain-wise overall Cronbach’s α was 0.805, which did not increase after deleting any domains. The corrected inter-domain total correlation coefficients were 0.520, 0.745, and 0.694 for IROD, DRDC, and ELID domains, respectively (Table 2).

**Table 2:** Questionnaire reliability

Domain	No. of Questions in each Domain	Cronbach’s Alpha if Domain Deleted	Corrected Domain Total Correlation
IROD*	10	0.835	0.520
DRDC^†^	14	0.703	0.745
ELID^‡^	9	0.753	0.694

* Increase rate of organ donation

† Decrease rate of donation related crime

‡ Ethical and legal issue related with donation

Test-Retest Reliability

Test-retest was done in the same participants at a 20-day interval and intra-class correlation coefficient was calculated for each question (Table 3).

**Table 3:** Test-retest reliability

Question	Intra-class Correlation Coefficient* (95% CI)
1	0.664 (0.529–0.759)
2	0.531 (0.302–0.678)
3	0.635 (0.468–0.746)
4	0.744 (0.621–0.824)
5	0.808 (0.705–0.871)
6	0.808 (0.735–0.861)
7	0.698 (0.562–0.788)
8	0.593 (0.442–0.704)
9	0.584 (0.425–0.699)
10	0.895 (0.854–0.925)
11	0.675 (0.523–0.774)
12	0.616 (0.377-0.751)
13	0.747 (0.641–0.821)
14	0.728 (0.600–0.811)
15	0.698 (0.573–0.785)
16	0.726 (0.553–0.823)
17	1
18	1
19	0.768 (0.600–0.855)
20	0.726 (0.570–0.818)
21	0.844 (0.756–0.896)
22	0.740 (0.635–0.813)
23	1
24	0.730 (0.628–0.804)
25	0.700 (0.566–0.790)
26	0.751 (0.639-0.826)
27	0.621 (0.479–0.724)
28	0.771 (0.657–0.844)
29	0.738 (0.606–0.822)
30	0.698 (0.579–0.783)
31	0.627 (0.448–0.742)
32	0.614 (0.456–0.724)
33	0.630 (0.481–0.734)

* p<0.01

Factor Analysis

Factor analysis was done for total scores obtained in each domain using principal component analysis with direct oblimin rotation. Only one factor (i.e., organ donation) was present in this study that was supported by examination of eigenvalue and scree plot. Examination of data indicated the sample was factorable (Kaiser-Meyer-Olkin measure of sampling adequacy was 0.642); Bartlett’s test of sphericity was significant (p<0.001).

## DISCUSSION

Organ transplantation, a life-saving treatment, is not a well-established service in South-Asian countries like Nepal due to religious, cultural, and legislative barriers rather than lack of resources or optimal infrastructure. Currently, the trained men-powers in the field of organ transplantation are capable of managing the transplant service in our country but there is a lack of clear laws and regulations supporting the diverse cultural and religious views of our population, which is the main obstacle for the development of transplant service in Nepal. This gap can only be fulfilled by collecting and including the public views related to organ donation in our regulatory system. The public views can be collected through the questionnaire developed in this research. After reviewing the regulatory systems of the countries with increased rate of donation, ten questions were developed asking whether those systems like presumed consent, active detection, and modified version of presumed consent, paired exchange, list exchange, and donor compensation can be incorporated in our regulations. The adoption of such system requires addressing ethical dilemma associated with them. The World Medical Association (WMA) ensures that its ethical policy statements reflect a consensus by requiring a 75% vote in favor of any new or revised policy at its annual assembly [17]. Similarly, to collect public consensus, in order to solve the ethical dilemma, nine questions on presumptivity, opt-out decision and so on were developed. After reviewing our current laws on organ transplantation and discussing with the experts regarding shortcomings in our laws, which have encouraged the organ trading, fourteen questions were added to address these issues. 

A question regarding the priority for receiving organ was also developed, as patients who are in terminal stage at the time of liver transplantation have inferior survival rates compared to those who are transplanted in a better physiological state [18, 19]. The debate should probably take place at the level of the population, which, in a democracy, should be in charge of these fundamental issues that deal with the therapeutic relationship, the role of medicine, and the status of the individual as a human being. The development of the questionnaire provided an opportunity for taking the debate beyond the health care community and for ensuring transparency of all transplantation activities in general and also for organizing an exhaustive information campaign with all the learned societies involved, the authorities responsible for regulating transplantation activities, organ retrieval, and transplantation networks, patients on transplantation lists, health care professionals involved with transplantation, legislators, and the general public, to clarify issues raised by organ retrieval and the conduct of procedures for organ retrieval, including the legal, technical, and moral requirements. Good communication with and guidance of the family throughout the end-of-life care and donation procedure is of utmost importance for their acceptance of donation and bereavement care [20].

The strength of the questionnaire developed was that it was found valid and reliable. The reliability testing was done without any interaction, which showed that the questionnaire is not complicated, can be self-administered, can avoid some potential for observer bias and needs no special training to administer the test. All 153 participants tested also participated in retest, done after 20 days; the intra-class correlation coefficient between test and retest results for each question was moderate to strong (positively correlated). Kaiser-Meyer-Olkin measure above 0.6 and significant Bartlett’s test of sphericity indicated that the sample size was adequate for factorable; examination of eigenvalue and scree plot further supported our single factor measurement, i.e., organ donation. 

Each question in the questionnaire is preceded by some information, which would act as the campaign for encouraging organ donation. Convergent validity could not be performed on the questionnaire as we could not find a similar study with which we could correlate the results. Discriminant validity was also not done because of simplicity of the questionnaire whose responses were not affected by presence or absence of participant’s knowledge regarding organ transplantation.

In conclusion, a valid and reliable questionnaire was developed that can be used to achieve public, and health professionals consensus on the most appropriate and the most acceptable regulating system of organ transplantation. This questionnaire is recommended for researchers from different countries of the world so that the opinions thus gathered will be of relevance to their respective nations and may provide a guide for the policy makers regarding organ transplantation system.
